# Data Import and Validation in the Inorganic Crystal Structure Database

**DOI:** 10.6028/jres.101.039

**Published:** 1996

**Authors:** H. Behrens

**Affiliations:** Fachinformationszentrum Karlsruhe, D-76344 Eggenstein-Leopoldshafen, Germany

**Keywords:** bibliometrics, crystal structures, databases, data checking, data recording, data import, ICSD, inorganic compounds

## Abstract

In the following paper the input procedures for the Inorganic Crystal Structure Database (ICSD) will be outlined. The input flow of the data is explained. Since the data have been excerpted from journal articles a bibliometric analysis of the relevant literature is presented. The types of data and the form in which they are recorded are discussed. Finally, illustrations are given of the importance of data checking and the data checking procedures are described in detail.

## 1. Introduction

This paper describes how data are selected, obtained, recorded and checked for the Inorganic Crystal Structure Database (ICSD), which was originally developed at the University of Bonn and is now produced by FIZ Karlsruhe and the Gmelin Institute.^1^ A general description of the ICSD database itself and of the corresponding retrieval tools has already been given by Prof. E. Fluck [E. Fluck, J. Res. Natl. Inst. Stand. Technol. 101, 217 (1996)] in his presentation. These will not be repeated.

For data to be included in ICSD, the following selection criteria are applied: Data are taken into account from all compounds which

- have no C—C and/or C–H bonds in any residue
- and contain at least one of the nonmetallic elements H(D), He, B, C, N, O, F, Ne, Si, P, S, Cl, Ar, As, Se, Br, Kr, Te, I, Xe, At, and Rn

As far as the data themselves are concerned the following metadata, bibliographic data, crystal structure data and related parameters as well as properties are included:

- compound designation
  chemical name
  chemical formula
  mineral name
- bibliographic data
  Title
  Authors
  citation
- data
  unit cell dimensions
  space group
  *atomic parameters:*
  element symbol with numbering
  oxidation state
  multiplicity and Wyckoff letter
  coordinates
  site occupation
  thermal parameters
  reliability index R

In the next sections details of the input flow paths, some bibliometric considerations concerning the journal articles from which the data are taken, and details of the data recording procedure will be given. Special emphasis is also laid on the validation of the data by automatic as well as manual checking. An earlier description of ICSD in this context can be found in Ref. [1].

## 2. Data Flow

**Fig. 1:**
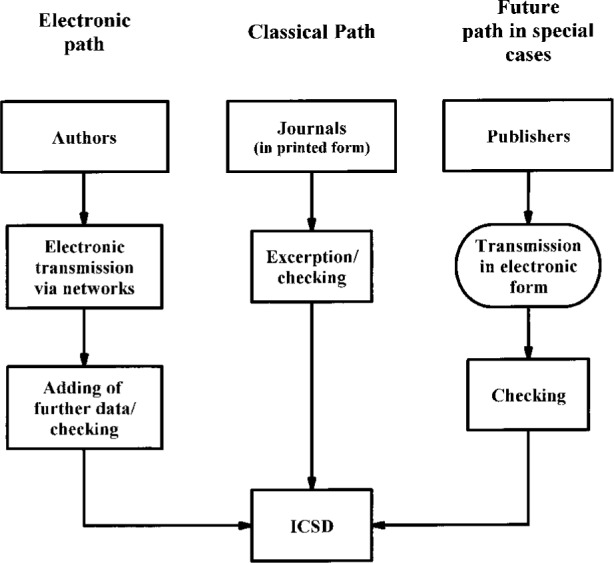
Schematic input flow diagram for ICSD.

The data flow is schematically represented in Fig. 1. Three paths are of importance in this context. First, the classical path, which is still the most important one. The overwhelming majority of the input still gets into the database in this way. This also means that the data stored in the database have been taken from journals. Secondly, we have a more modern path where the data are transmitted electronically by the authors. This way is a very new one and may be of more and more importance in the future. In this case, where the data are directly transmitted by authors there is no interruption (by printing and re-keying of the figures) in the data flow from the original measurement. Thirdly, in the near future we will receive data from publishers in electronic form. A first agreement has already been concluded with the IUC in this context.

**Fig. 2:**
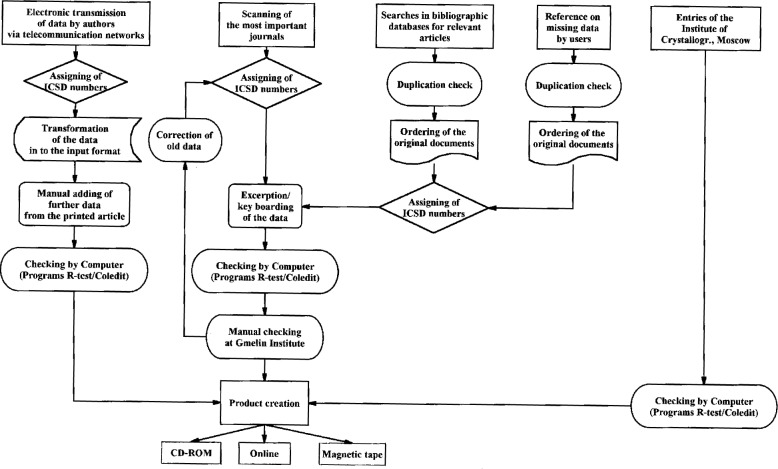
Detailed input flow diagram for ICSD.

A more detailed description of the input flow, which shows how the information is obtained for ICSD at present, is given in Fig. 2.

As mentioned before most of the data are taken from journal articles. Most of these are scanned in-house, the relevant articles are marked up, ICSD numbers are assigned to each entry, the data are excerpted and keyboarded. Then the data are checked by computer and manually. Finally the products (CD-ROM, online, magnetic tape) are created. For journals which do not contain so many articles with relevant data, searches in bibliographic databases are carried out, and the original documents are then ordered. The subsequent procedure is then the same as just explained. In some cases users inform us of missing data, which we then add. We also have a cooperation with the Institute of Crystallography of the Russian Academy of Science in Moscow. This institute delivers data to us in machine-readable form. The data are again checked at FIZ Karlsruhe.

**Fig. 3:**
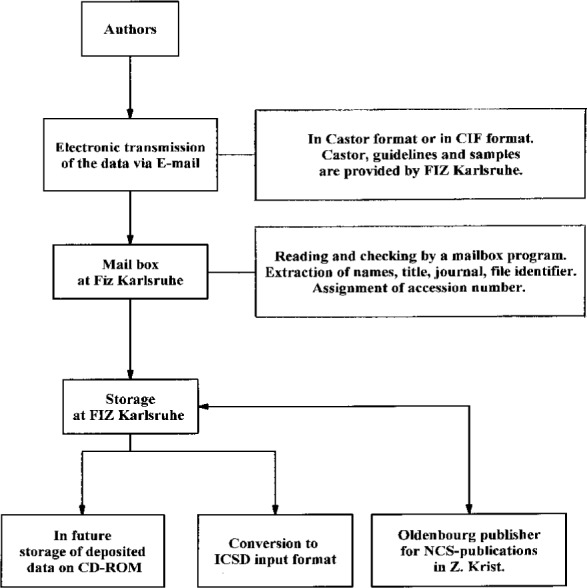
Electronic deposition of crystal structure data at FIZ Karlsruhe.

In the case of a number of journals data which are not printed are deposited at FIZ in electronic form. Details are shown in Fig. 3. These data are electronically transmitted by e-mail to a mailbox at FIZ by the authors via telecommunication networks (Internet) and stored at FIZ. The relevant data are selected and converted to ICSD input format for further processing. Further data, for instance the volume number and pages, are added manually. These data also have to pass the checking procedure mentioned above.

## 3. Bibliometrics

**Fig. 4:**
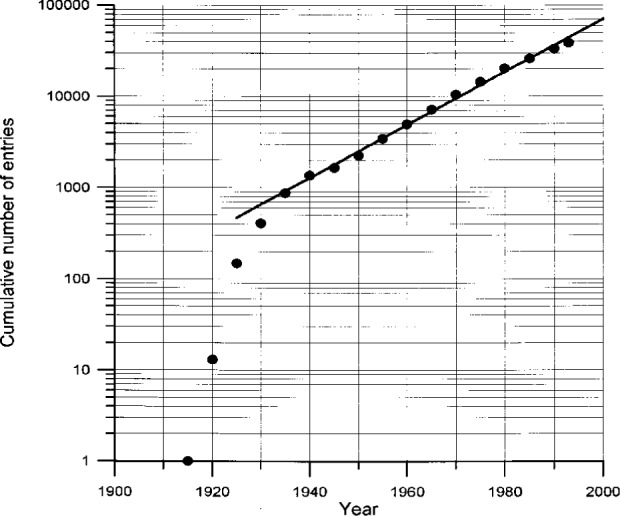
Cumulative number of entries of ICSD as a function of the publication year of the articles the data originate from (FIT exponential, doubling time 10.4 years).

As already said, practically all data originate from journal articles. Therefore it might be of some interest for prospective users to have a more detailed bibliometric analysis of the ICSD content. In Fig. 4 the development of the cumulative number of entries over time (publication year of the articles containing the data) is shown on a semilogarithmic scale. We immediately recognize the exponential growth of the total number of measured crystal structures in inorganic chemistry that exist up to now. The doubling time, which is 10.4 years, has nearly the same value as the doubling time for publications in physics and chemistry. By the way, the number of entries added to ICSD per year at present is about 2000.

**Fig. 5:**
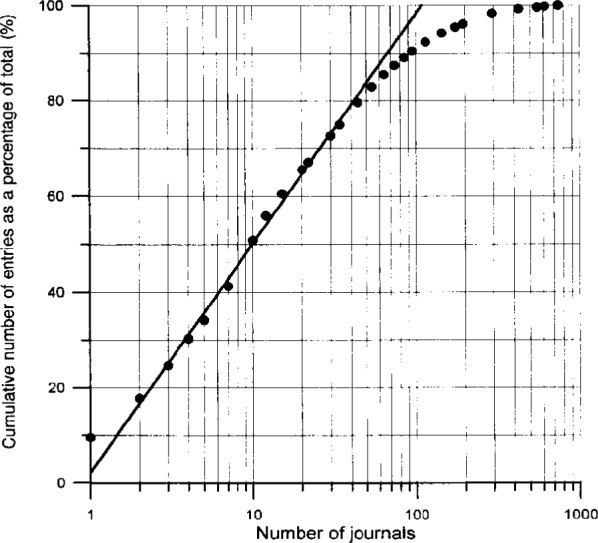
Bradford’s distribution for ICSD (Fit: *y* = 48.03 log *x* + 2.1562).

In the next (Fig. 5) the Bradford distribution for ICSD is presented. Here, the cumulative number of entries as a percentage of the total is plotted as a function of the number of journals containing the data. The journals are ranked in decreasing order of productivity. The scale is semilogarithmic. The total number of entries at present is 38 869. The following conclusions can be drawn from the Bradford distribution for ICSD.

The behavior in the log-linear range has the following form (fit):

y=48.03logx+2.1562 (1)

where

*y* = cumulative number of entries as a percentage of total number of entries in ICSD
*x* = number of journals

dy/dx=20.86(l/x) (2)

This means if one further journal is taken into account

for *x* = 3 6.95 % of entries is added to ICSD,
for *x* = 20 1.04 % of entries is added to ICSD,
for *x* = 50 0.42 % of entries is added to ICSD.

**Fig. 6:**
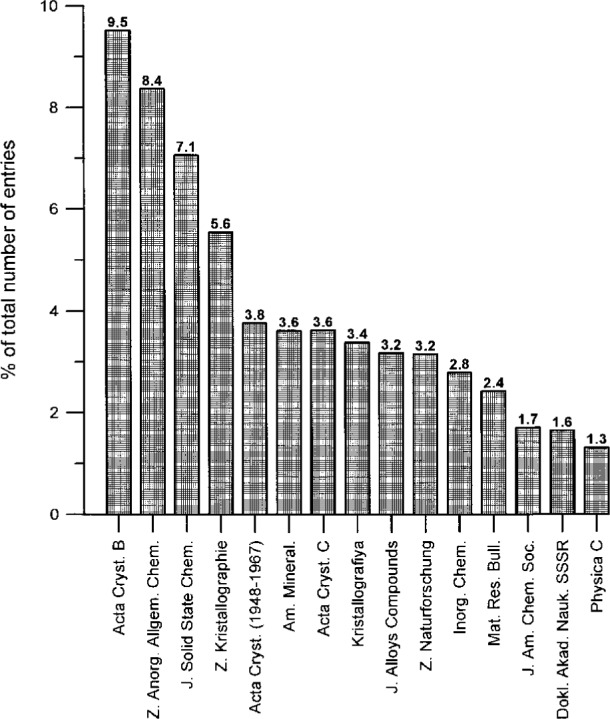
Percentage of the total number of entries of ICSD originating from the journals with the largest numbers.

**Fig. 7:**
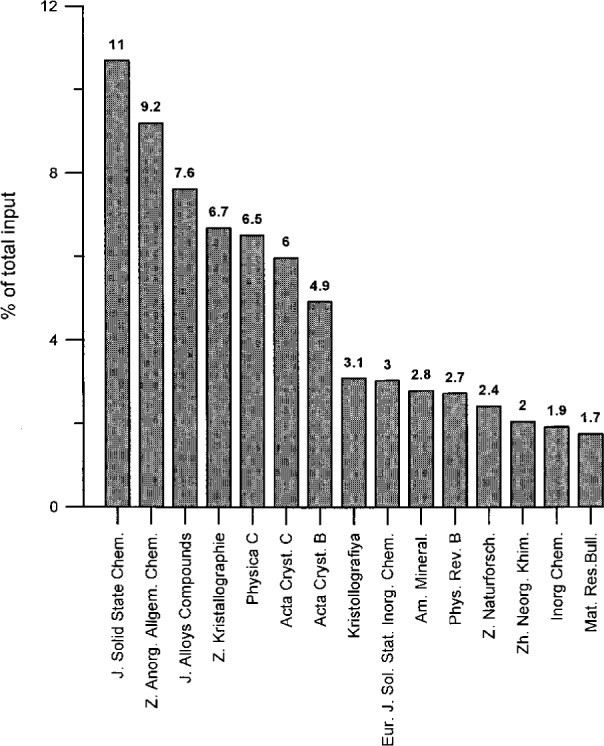
Percentage of total input of ICSD for the journals with the largest number of entries for the publication years 1990–1993 (total input = 7029 entries).

One also immediately recognizes that about 50 % of the entries come from only 10 journals. In Fig. 6, the 15 journals with the largest number of entries (together with the percentage contributions to the total number of entries) are explicitly represented as a bar chart diagram for the total content of the database (they already represent 61.2 % of the total content). Over the years, however, some changes occurred as far as the contribution of different journals is concerned. Therefore, in Fig. 7 the same diagram is shown for the input of entries originating from articles of the publication years 1990–1993 in order to describe the situation which we have today (they already represent 71.5 % of the total input for these years). At present the input per year for ICSD originates from 100–200 journals. Of these, 21 journals are regularly scanned at FIZ Karlsruhe.

**Fig. 8:**
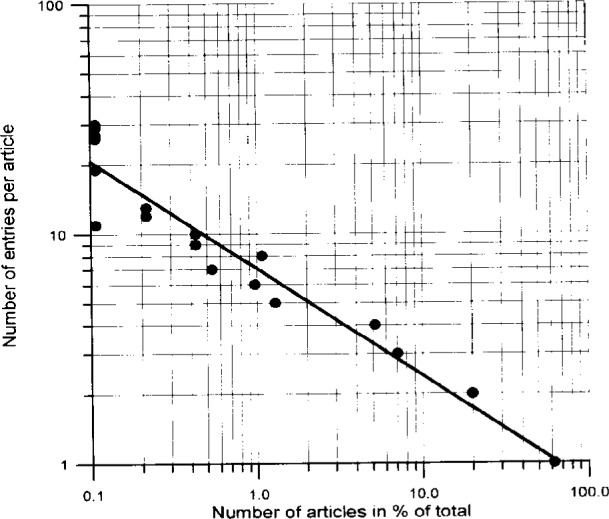
Number of entries per article of ICSD as a function of the number of articles the data originate from for the publication year 1992.

**Fig. 9:**
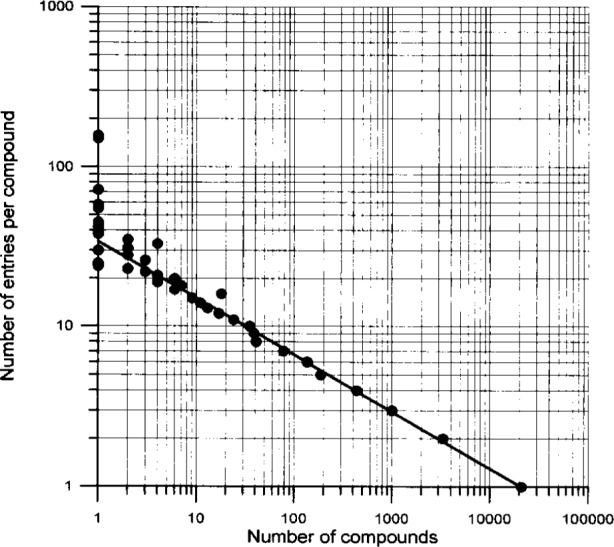
Number of entries per compound of ICSD as a function of the number of compounds (Total number of compounds = 26478).

For input considerations it might be also of interest to have some information on the number of entries per journal article. Thus, Fig. 8 shows how many articles contain how many entries for the publication year 1992. For example, one sees that about 60 % of the articles contain only one entry. In a number of cases one compound has been investigated several times. Therefore, more than only one entry per compound is contained in the database for a number of compounds. This is demonstrated in Fig. 9.

## 4. Data Recording

How and in what form the data are recorded will be elucidated in the following. This can best be done by explaining the input record structure in detail. Here, all fields are listed which make up an input record. Then, the field contents are described, followed by an example for each case. In fields 7 and 9, the numbers following each ± symbol represents the estimated standard deviation. Such an input record consists of the following fields:

1. Name field
  Tag: N
  Content: Name in English according to IUPAC rules with oxidation state where necessary
  Example:
  N xxxxxx 1 Iron(III) hydroxide sulfate
2. Mineral name field
  Tag: M
  Content: Mineral name, including details of origin following '-'
  Example:
  M xxxxxx 1 Cryolite—synthetic
3. Chemical formula field
  Tag: F
  Content: Formula using only accepted symbols (iodine = I, (deuterium = D)
  Example:
  F xxxxxx 1 Ca (H2 P O4)2 (H2 O)2
4. Title field
  Tag: U
  Content: Title of publication in original language if possible, otherwise in English as written in original publication
  Example:
  U xxxxxx 1 The crystal structure of Na_2_(SO_4_)
  U xxxxxx 1+1 A simple structure
5. Citation field
  Tag Q
  Content: Citation, i.e., year and coden (with check-letter) for journal, volume, first page, last page and issue number if necessary.
  Example:
  Q xxxxxx 1 81ZSTKAI 22 1 5 3
6. Authors fields
  Tag: A
  Content: Authors, i.e, surname followed by the initials only, not forenames
  Example:
  A xxxxxx 1 Mueller H
7. Unit cell field
  Tag: E
  Content: Unit cell, i.e., *a, b, c* (in Å), *α*, *β*, *γ* (max. 7 characters each), number of formula units *Z* (integer of max. 3 characters), measured density (max. 6 characters), all with standard deviations except *Z*.
  Example:
E xxxxxx1  5.638±3 7.639±4 13.25±490. 90. 90. 2 4.53±2
8. Space group field
  Tag: R
  Content: Hermann-Mauguin space group symbol
9. Atomic parameter fields
  Tag: P
  Content: Atomic parameters, i.e.,
  1. Element symbol
  2. Atom identifier for this atom
  3. Oxidation state (max. 5 characters)
  4. Multiplicity and Wyckoff letter
  5.–7. *x y z* (max. 7 characters each)
  8. Isotropic displacement (temperature) factor B or ‘U’ or ‘*’ if no displacement factor is given or ‘B’ if anisotropic displacement factors follow on B, C, or D records (max. 6 characters).
  9. Site occupation (max 5 characters).
  10. Number of H atoms bonded to this atom, followed directly by ‘H’ or ‘D’
    Example:
P xxxxxx 1 O1  −2  2I  0.123±1  0.1231±1 0.5364±2  3.5±1  1  2HP xxxxxx 2 Fe 1  2.75  4G  0.25  0.25 0.45678±3  B  0.975P xxxxxx 3 Cl 1  −1  4G  0.25  0.25 0.2345±7P xxxxxx 4 Se 1  −2  4G  0.25  0.25 0.1497±1  U0.012±1
10. Anisotropic displacement factors fields
  Tag: B
  Content: Anisotropic displacement factors if given a *β* (max. 8 characters each).
  Example:
B xxxxxx 1O1  0.00234±2  0.0025±1  0.00024±1  −0.0005±2  −0.00032±2B xxxxxx 1+1  0.0003±2
11. Anisotropic displacement factors fields
  Tag: C
  Content: Anisotropic displacement factors if given as *B* (max. 8 characters each).
12. Anisotropic displacement factors fields
  Tag: D
  Content: Anisotropic displacement factors if given as *U* (max. 8 characters each).
13. Remarks field
  Tag: Z
  Content: Remarks (Each remark is standardised and has a separate record).
  Example: XDP (x-ray diffraction of powder)
14. R-index field
  Tag: I
  Content: R-index (max. 5 characters) Example: 0.05
15. Test code field
  Tag: Y
  Content: Test code to be added during checking procedure
  Example: 53 (at least one temperature factor missing in the paper)

xxxxx represents the special COL-number of the entry under consideration. For didactic reasons the contents are decoded in some way. Standard deviations are connected with a + sign only. An example of an input record is given in Table 1. The following software is used in the context of data recording: For administration a specially developed program (literature acquisition, duplication check, input status), for keyboarding SPF and Coledit, and for data checking R-Test and Coledit. The software makes the recording easy by applying predefined masks for the fields in which the data must be entered.

## 5. Data Checking

**Fig. 10:**
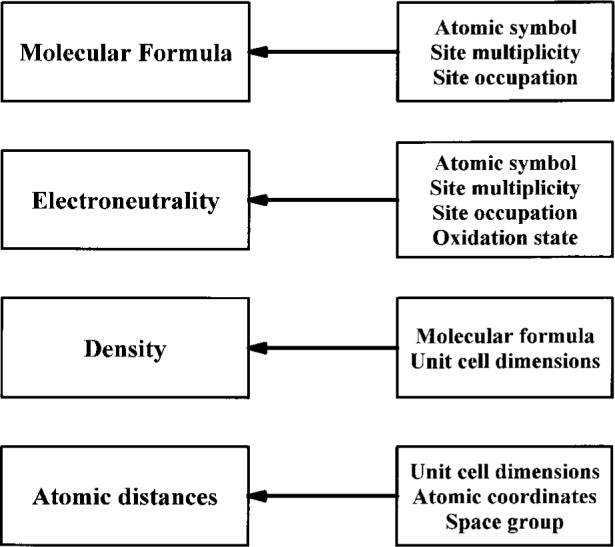
Schematic representation of the data checking for ICSD by requiring the consistency of redundant data.

Data validation is a very important, even essential point in the whole input procedure. Various careful data checks have to be taken into consideration in this context. Here, in a first step, data checking by computer is applied as far as possible. For this purpose, use is made of formal checking procedures, of plausibility considerations, of constraints following from mathematical and physical laws and of the fact that redundant data have to be consistent. The latter point is illustrated in Fig. 10 where the most important relations which are used for checks are summarised.

In detail the data checking by computer looks as follows:

- formal checking of
  - Correct structure
  - Missing field contents
  - Plausibility
  - Duplication
  - Syntax
- Verification of contents by checking of
  - Plausibility of cell
    2Å < *a*, *b*, or *c* < 80 Å
    10° < *α*, *β* or *γ* < 170°
  - Validity of cell
    *α* + *β* + *γ* ≤ 360°
    *α* ≤ *β* + *γ* ; *β* ≤ *α* + *γ* ; *γ* ≤ *α* + *β*
  - Plausibility of density
    0.5 g/cm^3^ < ρ < 20 g/cm^3^
  - Matching of cell and space group
    cub. *a*=*b*=*c*; *α*=*β*=*γ*=90°
    trig. *a*=*b*=*c*; *α*=*β*=*γ*≠90°
    tet. *a*=*b*≠*c*; *α*=*β*=*γ*=90°
    hex. *a*=*b*≠*c*; *α*=*β*=90°; *γ*=120°
    orth. *a*≠*b*≠*c*; *α*=*β*=*γ*=90°
    mon. *a*≠*b*≠*c*; *α* =*γ*=90° or *α*=*β*=90°
    tric. *a*≠*b*≠*c*; *α*≠*β*≠*γ*
  - Validity of oxidation state
    −4 < oxid. state < + 8
  - Validity of site occupation
    0 < site occup. ≤ 1.0
  - Plausibility of isotropic temperature factors
    0.2 < B < 15.0
    0.001 < U < 0.3
  - Plausibility of anisotropic temperature factors
factors 11 22 33 12 13 230.0001< *β* < 1.0 –1.0 < *β* < 1.00.5 < B < 15.0 –15.0 < B < 15.00.001 < U < 0.3 –0.3 < U < 0.3
  - Validity of multiplicity
    The multiplicity is adjusted to the coordinates.
    Then it is checked for consistency.
  - Plausibility of interatomic distances
    The distances are calculated on the basis of the atomic coordinates and of cell parameters, and are then compared with the distances estimated from the ionic radii of atoms.
  - Validity of electroneutrality
    The total charge must be zero.
  - Validity of molecular formula
    The molecular formula is calculated from atomic parameters, site occupation and site multiplicity, and compared with the corresponding formula given by the author.
  - Comparison of calculated and measured densities
    The density calculated on the basis of molecular formula and unit cell dimension must agree with the measured density within certain limits.

In the process of data checking by computer all errors detected by the applied software programs (R-Test, Coledit) are corrected as far as possible. An example of an input record with the corresponding checking diagnostics is shown in Table 1. In some cases, however, uncertainties remain which cannot be resolved during the input process. Then, the test flags set by the program are stored in the database assigned to the corresponding entries. Examples for such test flags with their occurrence in the database are:
Test flagOccurrence• Deviation of the charge sum from zero tolerable.1250• Calculated density unusual but tolerable.4247• Temperature factor implausible but agrees with the paper.4626• At least one temperature factor missing in the paper.11017• A site occupation is implausible but agrees with the paper.163• Lattice parameters are unusual but agree with the paper.110• Coordinates are those given in the paper but are probably wrong.652• Reported coordinates contain an error. Values corrected.124• Interatomic distances appear to be too short.12314

As one can easily see, these test flags contain certain warnings which might be very useful in some cases.

Last but not least, it should also be mentioned in this context that some information stored in the database is automatically generated by computer by making use of the data already entered. These are the so-called implicit descriptors which are listed in the following:

- Crystal system (SYST)
- Laue class (LAUE)
- Crystal symmetry (SYPR)
- Crystal class (CLAS)
- Pearson symbol (PRS)
- Formula type (ANX)

An example of these implicit descriptors for copper(I) chloride looks as follows:
*• SYST**cubic**• LAUE**m3m**• SYPR**NCENNPOL**• CLAS**–43m (Hermann Mauguin) –Td (Schoenflies)**• PRS**cF8**• ANX**AX*

Of course, in the database the implicit descriptors can be searched for in the same way as the other data.

It is very clear that data checking by computer always requires manual checking in addition. For ICSD the manual checking consists of

1. checking of the relevance of the special entry
2. checking of the chemical nomenclature (according to the IUPAC rules)
3. checking of the mineralogical nomenclature (according to the IMA convention) and of the phase designations
4. special evaluation of the diagnostics of the checking programs for the following topics
  - oxidation state
  - space group
  - unit cell parameters
  - atomic coordinates
5. further checking of
  - bibliographic records
  - formula structure
  - site occupations
  - remarks

In conclusion the prospective user may now have an impression of the input policy of the ICSD database. Input policy always has also to find the right balance between cost-effectiveness and quality of a database as determined by completeness, accuracy and actuality. I have tried to demonstrate what kind of efforts are made for the ICSD database.

## Figures and Tables

**Table 1:** Input record of ICSD with checking diagnostics. The numbers following each ± symbol is the estimated standard deviation

N 066693 1	Lithium tetrafluoroindate
F 066693 1	Li (In F4)
U 066693 1	Structure of lithium tetrafluoroindate
Q 066693 1	92ACSCEE 48 769 771
A 66693 1	Gravereau P
A 066693 2	Chaminade J P
A 066693 3	Gaewdang T
A 066693 4	Gannec J
A 066693 5	Pouchard M
A 066693 6	Hagenmuller P
E 066693 1	4.752±1 11.721±4 4.971±1 90 90 90 4 4.67
R 066693 1	Pbcn
P 066693 1	Li 1 1 4C 0 .0561±7 .25 1.14±11
P 066693 2	In1 3 4C 0 .33113±2 .25 B
P 066693 3	F 1 −1 8D .2620±3 .4423±2 .4308±4 B
P 066693 4	F2 −1 8D .2272±4 .1928±2 .4199±4 B
D 066693 1	1n1 .0049±1 .0073±1 .0064±1 0 .0000±1 0
D 066693 2	F1 .0122±7 .0125±7 .0116±7 −.0048±6 −.0035±7 −.0010±7
D 066693 3	F2 .0100±7 .0123±7 .0110±7 .0015±6 −.0044±7 .0006±7
Z 066693 1	TEM 298
I 066693 1	0.0144
	Diagnostics for Col = 066693, Rel = 0
DB056304	Warning Record E 1 Calc. dens. > meas. dens. (1.5 %)

## Footnotes

1 Certain commercial equipment, instruments, or materials are identified in this paper to foster understanding. Such identification does not imply recommendation or endorsement by the National Institute of Standards and Technology, nor does it imply that the materials or equipment identified are necessarily the best available for the purpose.
